# Mechanisms of Action And Clinical Implications of MicroRNAs in the Drug Resistance of Gastric Cancer

**DOI:** 10.3389/fonc.2021.768918

**Published:** 2021-11-29

**Authors:** Ying Liu, Xiang Ao, Guoqiang Ji, Yuan Zhang, Wanpeng Yu, Jianxun Wang

**Affiliations:** ^1^ Institute for Translational Medicine, The Affiliated Hospital of Qingdao University, Qingdao Medical College, Qingdao University, Qingdao, China; ^2^ School of Basic Medical Sciences, Qingdao Medical College, Qingdao University, Qingdao, China; ^3^ Clinical Laboratory, Linqu People’s Hospital, Linqu, China

**Keywords:** microRNA, gastric cancer, drug resistance, biomarker, therapeutic target

## Abstract

Gastric cancer (GC) is one of the most common malignant tumors of digestive systems worldwide, with high recurrence and mortality. Chemotherapy is still the standard treatment option for GC and can effectively improve the survival and life quality of GC patients. However, with the emergence of drug resistance, the clinical application of chemotherapeutic agents has been seriously restricted in GC patients. Although the mechanisms of drug resistance have been broadly investigated, they are still largely unknown. MicroRNAs (miRNAs) are a large group of small non-coding RNAs (ncRNAs) widely involved in the occurrence and progression of many cancer types, including GC. An increasing amount of evidence suggests that miRNAs may play crucial roles in the development of drug resistance by regulating some drug resistance-related proteins as well as gene expression. Some also exhibit great potential as novel biomarkers for predicting drug response to chemotherapy and therapeutic targets for GC patients. In this review, we systematically summarize recent advances in miRNAs and focus on their molecular mechanisms in the development of drug resistance in GC progression. We also highlight the potential of drug resistance-related miRNAs as biomarkers and therapeutic targets for GC patients.

## Introduction

Gastric cancer (GC) is one of the most common malignant diseases of the digestive tract and the third leading cause of cancer-related deaths worldwide (1). According to global cancer statistics, GC ranks fifth for incidence and fourth for mortality among all types of cancer, with more than one million new cases and an estimated 769,000 deaths occurring in 2020 (2). Although the incidence of GC is declining gradually, it is still a major public health problem that seriously threatens patients’ health and lives (3). Currently, common treatment approaches to GC include chemotherapy, radiation, surgery, and targeted therapies. Depending on the resectability, stage, and status of GC patients, these therapies can be used in combination to improve their survival and life quality. However, for metastatic GC, chemotherapy is the main treatment method since most advanced patients fail to benefit from surgical resection or radiotherapy (4).

Chemotherapy is the first-line standard treatment option for all stages of cancer and can effectively delay or avoid cancer recurrence in the short term. However, the long-term role of chemotherapy in extending patient survival is very limited (5). One of the main reasons is the development of drug resistance, which results in chemotherapy failure, cancer recurrence, and finally patient death. It has been reported that drug resistance is correlated with more than 90% of cancer-related death (6, 7). Overall, drug resistance can be classified into two categories: intrinsic and acquired. Its underlying mechanisms are very complicated, mainly including changes in drug efflux, the inhibition of cell apoptosis, alterations of the cell cycle, enhancement of DNA damage repair, mutations of drug target genes, and the dysregulation of epithelial mesenchymal transformation (EMT) as well as the acquisition of cancer stem cell (CSC) properties (8). However, the detailed mechanisms involved in drug resistance are still unclear.

MicroRNAs (miRNAs) are a large group of small ncRNAs involved in practically all major biological processes *via* the direct post-transcriptional inhibition of target mRNAs. It has been reported that approximately 2600 miRNAs molecules have been identified in the human genome, and more than 60% of human protein coding genes are regulated by miRNAs (9, 10). MiRNAs can simultaneously regulate multiple target genes involved in different cellular processes, such as signal transduction, cell differentiation, apoptosis and proliferation (11). Therefore, the dysregulation of miRNAs contributes to many pathological processes, including GC. Moreover, the aberrant expression of miRNAs has been observed in GC (12). A growing amount of evidence suggests that miRNAs may play crucial roles in the drug resistance of GC. In this review, we provide a brief description of recent findings regarding the biogenesis and functions of miRNAs and highlight their underlying mechanisms in the drug resistance of GC.

## Overview: Biogenesis and Functions of MiRNAs

### Biogenesis of MiRNAs

**Figure 1:**
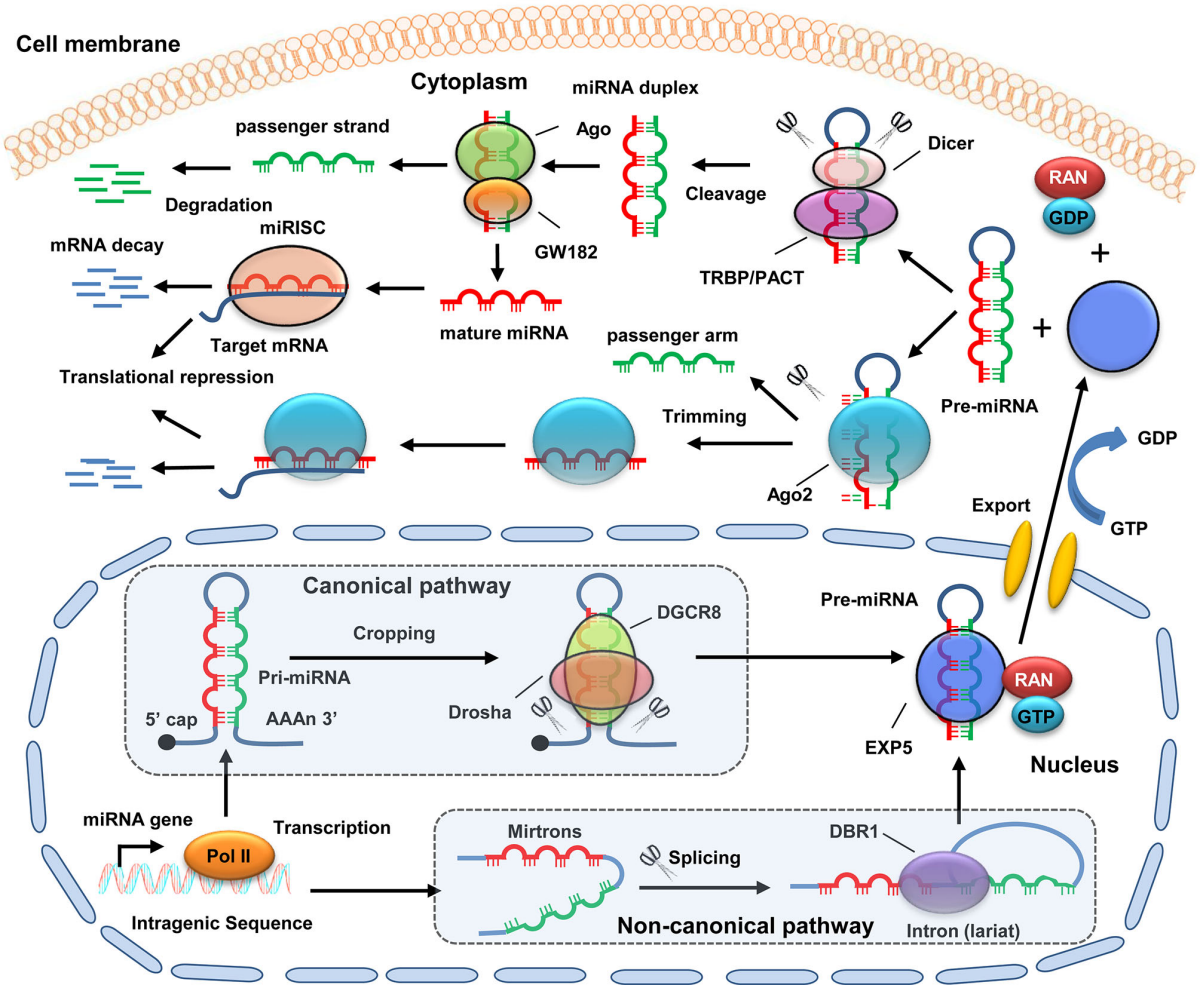
Schematic diagram of miRNA biogenesis. For canonical pathway, pri-miRNA is transcribed by Poll II from original miRNA genes in the nucleus. Then, pri-miRNA is processed into pre-miRNAs by a microprocessor complex consisting of Drosha and DGCR8. For non-canonical pathway, mirtron is transcribed from original miRNA genes. Then, mirtrons undergo splicing to form a lariat structure, which is further processed into pre-miRNAs by DBR1. Next, the pre-miRNAs are exported to the cytoplasm with the help of EXP5, GTP, and RAN. In the cytoplasm, the pre-miRNA is further recognized and processed into double-stranded miRNAs by the Dicer/TRBP/PACT complex. Subsequently, the double-stranded miRNAs are unwound into a guide strand and a passenger strand *via* Ago protein. The passenger strand is then degraded, whereas the guide strand is incorporated into RISC to form a mature miRNA. MiRNA binds to the 3’-UTR of mRNAs, and ten promotes its degradation. In addition, some pre-miRNAs (e.g., miR-451) can be cleaved by Ago2 to generate an intermediate 3’ end, which is then further trimmed.

MiRNAs are endogenous RNA-type molecules transcribed by RNA polymerase II (Pol II) with 19–25 nucleotides in length (13). The biogenesis mechanism of miRNAs has been well-studied. The majority of miRNAs are generated by the canonical biogenesis pathway (**Figure 1**). In this pathway, primary miRNAs (pri-miRNAs) transcribed from original miRNA genes are processed into precursor miRNAs (pre-miRNAs) in the nucleus by a microprocessor complex consisting of DiGeorge syndrome critical region 8 (DGCR8) and ribonuclease III enzyme Drosha. Subsequently, pre-miRNAs are exported to the cytoplasm through exportin 5 (EXP5) and then further recognized and processed into double-stranded miRNAs by the Dicer/TRBP/PACT complex. Next, the double-stranded miRNAs are unwound into a guide strand and a passenger strand *via* helicase. The passenger strand (with a higher stability) is subsequently degraded, whereas the guide strand (with a lower stability) is incorporated into the RNA-induced silencing complex (RISC) to form a mature miRNA (6). In addition, multiple non-canonical miRNA biogenesis pathways have been clarified. These non-canonical biogenesis pathways are group into Drosha/DGCR8-independent and Dicer-independent. For instance, the precursor stem lengths of some miRNAs are shorter than canonical pri-miRNAs, which can’t be recognized and processed by Drosha/DGCR8. These miRNA precursors are encoded in short introns that are named mirtrons. Mirtrons possess a hairpin structure and undergo splicing. The resulting products form a lariat structure and are processed into pre-miRNAs by DBR1 (lariat debranching enzyme), which are followed by further processing *via* Dicer (14). MiR-451 is a typical miRNA produced in a Dicer-independent manner. Its maturation does not require Dicer catalysis. The pre-miRNA of miR-451 is cleaved by Ago2 to generate an intermediate 3’ end, which is then further trimmed (15).

The biogenesis of miRNAs is modulated by various factors, including post-translational modifications, target mRNAs, RNA binding proteins (RBPs), and long non-coding RNAs (lncRNAs). For instance, DGCR8 can be modified by SUMO1. The SUMOylation of DGCR8 regulates its affinity with pri-miRNAs, leading to an alteration in the pri-miRNA functions of the recognition and repression of the target mRNAs (16). Bose et al. found that target mRNA promotes the biogenesis of its cognate miR-122 by enhancing the activity of AGO2-associated DICER1 (17). Moreover, Treiber et al. screened approximately 180 RBPs that interact specifically with different pre-miRNAs. Functional analysis showed that a large number of these RBPs, including splicing factors and other mRNA processing proteins, play a role in regulating miRNA processing (18). In addition, lncRNA NEAT1 is reported to modulate global pri-miRNA biogenesis by broadly interacting with the NONO–PSF heterodimer and many other RBPs (19). Collectively, the biogenesis of miRNAs is tightly regulated by diverse mechanisms. The dysregulation of miRNA biogenesis would contribute to many pathological progresses, particularly cancer progression.

### Functions of MiRNAs

MiRNAs exert their biological functions by negatively regulating the expression of their target mRNAs *via* directly binding to 3′ untranslated regions (UTR), leading to the inhibition of their translation (20). The precise regulation of miRNA expression and activity is crucial for maintaining common physiological conditions. MiRNAs are involved in the regulation of almost all major physiological and pathological processes, such as DNA damage, encompassing metabolism, apoptosis, differentiation, proliferation, and cell cycle as well as drug resistance (21–23). Therefore, any dysregulation of miRNA function and aberrant expression may lead to the occurrence of pathological events, particularly cancer. In fact, the abnormal expression of miRNAs has been observed in a number of cancer types, including GC. MiRNAs act as oncogenes or tumor suppressors to play crucial roles in cancer progression (24). Moreover, increasing evidence suggests that miRNAs are closely associated with drug resistance of GC, and some of miRNAs possess great potential as novel biomarkers and therapeutic targets in reversing drug resistance in GC (25). However, further studies are still required to elucidate the detailed mechanism of miRNAs in the drug resistance of GC.

## Implication of MiRNAs in GC Drug Resistance

**Figure 2:**
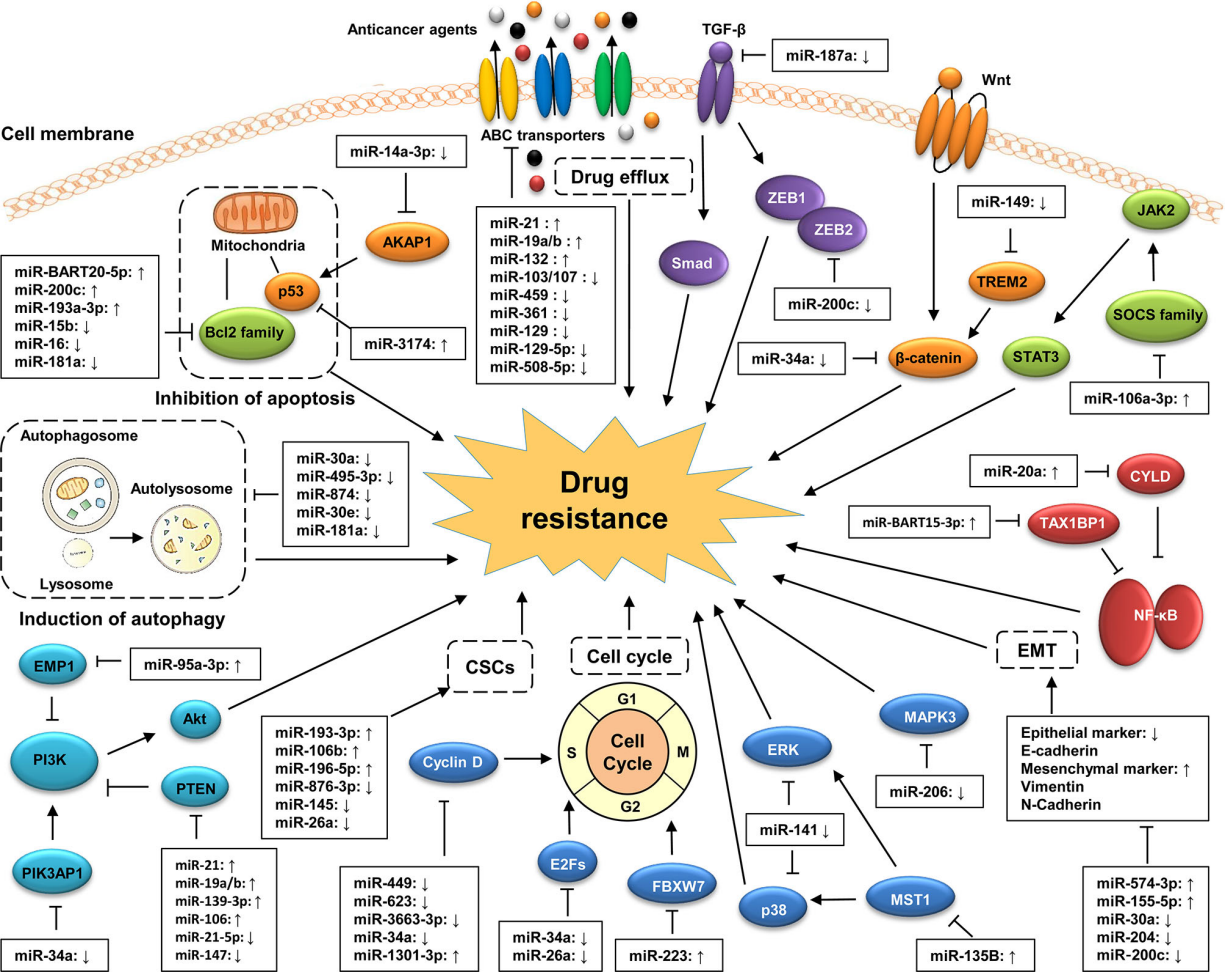
Classic mechanisms of miRNA involved in the drug resistance of GC. MiRNAs participate in the development of GC drug resistance by regulating multiple biological processes of GC cells, including drug efflux, cell apoptosis, cell cycle, and EMT as well as the acquisition of cancer stem cell (CSC) properties. MiRNAs also involves in the regulation of GC drug resistance by targeting cancer-related signaling pathways, such as the PI3K/Akt, Wnt/β-catenin, MAPK, TGF-β/Smad, and NF-κB signaling pathways.

Drug resistance is the most critical obstacle in GC effective treatment, blocking novel therapies and bringing about huge financial burden to patients and their families (26). The underlying mechanisms of drug resistance are very complicated and have not been fully elucidated. MiRNAs play crucial roles in the development of drug resistance in GC progression (**Figure 2**). For instance, miRNA expression profiles are associated with drug resistance (27), suggesting the potential of miRNA analysis as a valuable tool in precisely assessing the sensitivity of cancer cells to chemotherapy in GC treatment. MiRNAs participate in drug resistance by modulating the drug targets of multiple cellular pathways involved in the response to chemotherapy (28, 29). However, the detailed mechanisms of miRNA in drug resistance in GC are still inconclusive and are required to be elucidated.

### MiRNAs and Drug Resistance

Currently, the first-line chemotherapeutic agents for GC mainly include 5-fluorouracil (5-FU), doxorubicin (DOX), vincristine (VCR), platinum drugs, and paclitaxel (PTX). The involvement of miRNAs in GC resistance to these drugs is summarized in **Table 1**.

**Table 1:** MiRNA and drug resistance in GC.

Chemotherapeutic agent	miRNAs	Alteration	Effect on chemotherapy	References
5-FU	miR-149; miR-106a-5p; miR-421; miR-6785-5p; miR-130b; miR-147	Up	Reduction	(30–34)
	miR-195; miR-204; miR-567; miR-30a; miR-195-5P; miR-BART15-3p; miR-31; miR-623	Down	Enhancement	(35–42)
DOX	miR-223; miR-21-5p; miR-501; Lin28; miR-501; miR-663; miRNA-135a-5p; miR-92a; miR-520h	Up	Reduction	(43–51)
	miR-3064-5p; miR-217; miR-494; miR-495; miR-16-1	Down	Enhancement	(52–56)
VCR	miR-19a/b	Up	Reduction	(57)
	miR-101; miR-508-5p; miR-1284; miR-874; let-7b; miR-647; miR-126; miR-1; miR-23b-3p; miR-15b; miR-16; miR-200bc/429; miR-497; miR-181b	Down	Enhancement	(29, 58–69)
CDDP	miR-588; miR-522; miR-7; miR-4290; miR-21; miR-135b-5p; miR-379-5p; miR-95-3p; miR-142-3p; miR-99a-5p; miR-223-3p; miR-505; miR-500a-3p; miR-492;	Up	Reduction	(70–82)
	miR-34c; miR-34a; miR-152-3p; miR-30a-5p; miR-98-5p; miR-618; miR-1200; miR-let-7b; miR-30e; miR-4290; miR-3619-5p	Down	Enhancement	(73, 83–92)
OXA	miR-135a; miR-421	Up	Reduction	(93, 94)
	miR-567; miR-582-5p; miR-22-3p; miR-26; miR-361; miR-326; miR-3433-3p; miR-515-5p	Down	Enhancement	(37, 95–101)
PTX	miR-590-5p; miR-155-5p	Up	Reduction	(102, 103)
	miR-34c-5p; miR-21; miR-34a; miR-98; miR-34c; miR-217; miR-124-3p; miR-107; miR-495	Down	Enhancement	(46, 53, 55, 83, 87, 104–107)

#### MiRNAs and 5-FU Resistance

5-FU is a synthetic fluorinated pyrimidine analog with a fluorine atom at the C5 position in place of hydrogen. It shares a common structure with pyrimidine and can replace uracil to incorporate into RNA, leading to the disruption of RNA synthesis (108). Moreover 5-FU can also disrupt the intracellular deoxynucleotide pools by inhibiting thymidylate synthase (TS) required for DNA replication, thereby resulting in apoptosis and cell cycle arrest (109). 5-FU has been widely used to treat GC in clinic. However, the 5‐year survival rate of patients is still low due to the development of resistance to this chemotherapeutic agent. Multiple miRNAs are reported to be involved in the development of 5-FU resistance in GC. Several oncogenic miRNAs, such as miR-149, miR-130b, and miR-147, have been found to promote 5-FU resistance in gastric cancer (30, 33, 34). For instance, Wang et al. revealed that miR-149 confers 5-FU resistance by inhibiting TREM2 expression and modulating β-catenin pathway in GC cells (30). Similarly, miR-130b enhances the resistance of GC cells to 5-FU through targeting CMPK1 (33). By contrast, multiple tumor suppressor miRNAs, such as miR-195 and miR-204, can reverse 5-FU resistance of GC (35, 36). For instance, the overexpression of miR-195 enhanced the sensitivity of GC cells to 5-FU by downregulating HMGA1 expression (35). Exogenous expression of miR-204 sensitized GC cells to 5-FU by inhibiting EMT process *via* targeting TGFBR2 (36).

#### MiRNAs and DOX Resistance

DOX, also known as adriamycin (ADR), is an anthracycline and one of the more effective chemotherapy agents applied for GC treatment. Clinically, it is commonly used in combination with other chemotherapy agents, such as 5-FU, VCR, and PTX (27). DOX could exert strong cytotoxic effects on tumor cells through different molecular mechanisms including inhibition of DNA topoisomerase II, intercalation of DNA, and generation of free radicals (110). Multiple miRNAs have been shown to be responsible for the development of DOX resistance in GC. Several oncogenic miRNAs can promote DOX resistance in GC. For instance, miR-21-5p was highly upregulated in GC cell lines, and its overexpression increased DOX resistance in GC cells by inhibiting gene expression of phosphatase and tensin homologue (PTEN) and TIMP3 (44)(X21). Exosomal miR-501 could also significantly enhance DOX resistance by silencing cell death inducer (BLID), and, thus, suppressing caspase-9/-3 and p-AKT (45). Conversely, several tumor suppressor miRNAs can reverse DOX resistance of GC. MiR-16-1, for instance, has been found to increase the sensitivity of GC cells to DOX *via* inhibiting FUBP1 expression (56). The overexpression of miR-494 could also enhance the sensitivity of GC cells to DOX by directly targeting phosphodiesterases 4D (PDE4D) expression (54).

#### MiRNAs and VCR Resistance

VCR is an alkaloid extracted from vinca, which suppresses tubulin polymerization and disable spindles formation, thereby causing mitosis arrest of tumor cells (111). VCR is one of the first-line chemotherapy agents for GC treatment and often used in combination with other chemotherapeutic drugs. Multiple miRNAs have been reported to be involved in VCR resistance of GC. For instance, oncogenic miR-19a/b was highly expressed in VCR-resistant GC cells. The overexpression of miR-19a/b significantly enhanced the resistance of GC cells to VCR by directly targeting PTEN expression (57). On the contrary, some tumor suppressor miRNAs, such as miR-101, miR-126, and miR-181b, have been found to reverse the resistance of GC cells to VCR. Bao et al. showed that miR-101 was downregulated in GC tissues and cell lines. Forced expression of miR-101 could promote the sensitivity of GC cells to VCR by downregulating the expression of P-gp *via* targeting ANXA2 (58). Wang et al. revealed that miR-126 overexpression increased the sensitivity of GC cells to VCR *via* suppressing EZH2 expression (63). In addition, Zhu et al. found that ectopic expression of miR-181b could sensitize GC cells to VCR-induced apoptosis by downregulating BCL2expression (69).

#### MiRNAs and Resistance to Platinum Drugs

Platinum drugs are a class of cell cycle non-specific chemotherapeutic agent that widely used in clinical treatment of cancer patients. Currently, five platinum chemotherapy analogues have been approved for use in clinic, including cisplatin (CDDP), oxaliplatin (OXA), carboplatin, nedaplatin, and lobaplatin. These drugs directly insert platinum into DNA to form cross-links, which is either removed by specific DNA repair processes or it triggers a signaling cascade resulting in apoptosis of cancer cells (112). It has been reported that multiple miRNAs are involved in resistance to platinum drugs in GC. Several oncogenic miRNAs can facilitate the resistance of GC cells to platinum drugs. For instance, exosomal miR-588 from M2 macrophages increased the resistance of GC cells to CDDP *via* inhibiting the expression of cylindromatosis (CYLD) (71). In another study, exosomal miR-522 derived from cancer-associated fibroblasts (CAFs) was found to promote acquired resistance of GC cells to CDDP by inhibiting ALOX15 expression (70). Additionally, miR-135a was highly expressed in GC samples. The overexpression of miR-135a could promote the resistance of GC cells to OXA by inhibiting E2F1 and DAPK2 expression (93). By contrast, multiple tumor suppressor miRNAs can reverse platinum drugs resistance of GC. For instance, miR-34c overexpression was found to promote the sensitivity of drug-resistant GC cells to CDDP combined with paclitaxel by increasing the expression of apoptosis-related proteins. Consistent with this, inhibition of miR-34c significantly decreased the sensitization of drug resistant GC cells to CDDP combined with paclitaxel (83). Furthermore, the overexpression of miR-7 was able to enhance the sensitivity of GC cells to CDDP by inhibiting LDH-A expression (72). In addition, Qian et al. showed that miR-4290 was significantly downregulated in both GC samples and cell lines. Overexpression of miR-4290 could increase the sensitivity of GC cells to CDDP by suppressing PDK1-mediated glycolysis (73).

#### MiRNAs and PTX Resistance

PTX, a class of taxanes, is the most successful and widely used chemotherapeutic agent. As a natural anticancer drug, PTX promotes the assembly of tubulin into microtubules and resists the dissociation of microtubules, blocking mitosis progression, which resulting in programmed cell death (113). PTX is frequently used as the first-line treatment drug in GC. However, the resistance of GC to PTX is a great obstacle in its clinical applications. It has been reported that multiple miRNAs are involved in PTX resistance. Oncogenic miRNAs can promote PTX resistance. For instance, miR-590-5p was highly expressed in GC tissues and cells. Overexpression of miR-590-5p significantly decreased the sensitivity of GC cells to PTX by inhibiting RECK expression. Opposite result was obtained in miR-590-5p knockout GC cells (102). In another study, exosomal miR-155-5p derived from PTX-resistant GC cells has been found to promote drug resistance in PTX-sensitive GC cells by inhibiting the expression of GATA3 and TP53INP1 (103). On the contrary, several tumor suppressor miRNAs are able to reverse the resistance of GC cells to PTX. For instance, low expression of miR-34c-5p was observed in PTX-resistant GC tissues. Overexpression of miR-34c-5p significantly promoted the chemosensitivity of PTX-resistant GC cells (104). Tumor suppressor miR-21 has also been found to enhance the sensitivity of GC cells to PTX, at least in part, by modulating P-glycoprotein expression (105). In addition, overexpression of miR-34a and miR-98 could sensitize GC cells to PTX by suppressing the expression of E2F5 and BCAT1, respectively (87, 106).

### Expression Profiles of Drug Resistance-Related MiRNAs in GC

With the rapid development of detecting techniques and bioinformatics, a large number of miRNAs have been identified in GC. Many of them have aberrant expression levels and are closely correlated with the drug resistance of GC, indicating their great potential in predicting survival and response of patients to therapy (26). For instance, Sun et al. performed a microarray analysis to detect the miRNA expression profiles of GC cell-derived exosomes. They found that miR-106a-5p, miR-421, miR-19b-3p, miR-133a, and miR-214 are upregulated, whereas miR-144, miR-16-5p, miR-100, miR-30a-5p, and miR-361-5p are downregulated in MGC-803/5-FU exosomes compared with those in MGC-803 exosomes. High levels of miR-106a-5p and miR-421 in exosomes indicate 5-FU resistance in GC (31). Zhang et al. showed that 68 miRNAs were differentially expressed in SGC-7901/CDDP cells compared to SGC-7901 cells, including 41 upregulated miRNAs and 27 downregulated miRNAs. In BGC-823 and BGC-823/CDDP cells, 94 differently expressed miRNAs were observed, including 40 upregulated miRNAs and 54 downregulated miRNAs in BGC-823/CDDP cells. Among these differently expressed miRNAs, high levels of miR-99a and miR-491 indicate CDDP resistance in GC (114). In another study, using the Gene Expression Omnibus (GEO) database and GEO2R analysis, Zhou et al. confirmed 244 differentially expressed miRNAs in drug-resistant GC patients compared with in GC patients, among which 1120 were upregulated, and 124 were downregulated (115). Furthermore, Wei et al. reported 48 differentially expressed miRNAs (more than two-fold) in SGC7901/DDP cells when compared with SGC7901 cells, including 19 upregulated miRNAs and 29 downregulated miRNAs (116). Due to several factors, such as the heterogeneity of cancer cells, differences in research strategies, and differences in criteria for selecting significant miRNA profile data, the expression profiles of drug resistance-related miRNAs in GC may vary among studies. However, these studies still provide researchers with a new direction in showing that these altered miRNAs may be used to predict drug response to chemotherapy for GC patients.

### MiRNAs Regulate the Drug Resistance of GC by Targeting Signaling Pathways

An accumulating amount of evidence has shown that miRNAs are involved in the regulation of GC drug resistance by targeting cancer-related signaling pathways, such as the phosphatidylinositol 3-kinase (PI3K)/AKT, mitogen-activated protein kinase (MAPK), Wnt/β−catenin, nuclear factor kappa-light-chain-enhancer of activated B cells (NF-κB), and signal transducers and activators of transcription (STAT) signaling pathways. MiRNAs can change the chemotherapeutic sensitivity of GC *via* modulating the function or expression of some components of these pathways (117). Understanding the mechanisms of miRNAs in signaling pathway regulation may facilitate the development of new therapeutic strategies against GC drug resistance.

The PI3K/AKT signaling pathway is involved in the regulation of various cellular functions during cancer progression, such as proliferation, apoptosis, and metastasis (118, 119). This pathway also confers drug resistance to various types of cancer, including GC (120). Some miRNAs have been shown to mediate the drug resistance of GC by targeting the PI3K/AKT signaling pathway. For instance, Zhang et al. revealed that miR-567 enhances the sensitivity of GC cells to 5-FU and oxaliplatin. Mechanistically, miR-567 inhibits PI3K/AKT/c-Myc signaling pathway by blocking PIK3AP1 activity. Interestingly, c-Myc inversely regulates the expression of miR-567, leading to the formation of a miR-567-PIK3AP1- PI3K/AKT-c-Myc feedback loop (37). Ni et al. found that miR-95-3p activates the PI3K/AKT signaling pathway by upregulating the expression of p-PI3K and p-AKT, leading to the enhancement of CDDP resistance of GC (76). PTEN is a key negative regulator of the PI3K/AKT signaling pathway (121). Shen et al. showed that miR-147 is upregulated in GC tissues and cell lines. MiR-147 decreases the sensitivity of GC cells to 5-FU by targeting PTEN to activate the PI3K/AKT signaling pathway (34). In addition, high levels of exosomal miR-21 derived from M2 macrophages have also been demonstrated to facilitate CDDP resistance in GC cells by enhancing the activation of the PI3K/AKT signaling pathway *via* the downregulation of PTEN (74).

The MAPK signaling pathway is a classical carcinogenic pathway, and its aberrant activation has been shown to promote the initiation and progression of many cancer types, including GC (122, 123). It has been reported that some miRNAs contribute to the drug resistance of GC by regulating the expression of key components in the MAPK signaling pathway. For instance, Chen et al. found that low expression of miR-206 is closely correlated with the CDDP resistance of GC cells. The overexpression of miR-206 inhibits the proliferation of drug-resistant GC cells and decreases CDDP resistance by downregulating the expression of MAPK3 and p-MAPK3 (124). Another study showed that the downregulation of miR-135b enhances the CDDP sensitivity of GC cells. Mechanistically, miR-135b increases the expression of p-p38MAPK p38MAPK, p-ERK1/2, and ERK1/2 by targeting mammalian ste20-like kinase 1 (MST1), leading to the activation of the MAPK signaling pathway (125). Moreover, miR-20a is reported to promote the multidrug resistance (MDR) of GC cells by activating the epidermal growth factor receptor-mediated PI3K/AKT and MAPK/ERK signaling pathways by targeting LRIG1 (126). In addition, miR-27a-5p has been shown to enhance the sensitivity of GC cells to DOX by inhibiting the MAPK and AKT signaling pathways by targeting apurinic/apyrimidinic endodeoxyribonuclease 1 (APEX1) (127).

The Wnt/β−catenin signaling pathway plays crucial roles in maintaining the fundamental function of cells. Dysregulation of the Wnt/β−catenin signaling pathway has been widely observed in multiple types of cancer, including GC. Wang et al. showed that the overexpression of miR-149 enhances the 5-FU resistance of GC cells by activating the Wnt/β−catenin signaling pathway by targeting TREM2 (30). In another study, Chen et al. revealed that miR-34a mediates the enhancement of lncRNA HOTAIR on the CDDP resistance of GC cell lines by inactivating the Wnt/β−catenin signaling pathway (128). MiRNAs can also modulate the drug resistance of GC by targeting the transforming growth factor (TGF)-β signaling pathway. For instance, the expression of miR-187 is negatively associated with the CDDP-resistance of GC cells. The overexpression of miR-187 inhibits CDDP resistance in GC cells by downregulating the expression of TGF-β1 and p-Smad4 to inactivate the TGF-β/Smad signaling pathway (129). Additionally, miR-200c overexpression decreases the resistance of GC cells to trastuzumab by suppressing the TGF-β/Smad signaling pathway *via* downregulating zinc finger E-box-binding homeobox 1 (ZEB1) and ZEB2 expression (130). In addition, miR-362 and miR-20a have been reported to activate the NF-κB signaling pathway and upregulate the expression of NF-κB-regulated genes by targeting CYLD, leading to the enhancement of the CDDP resistance of GC cells (131, 132). In a study by Guo et al., miR-106a-3p was found to increase the apatinib resistance of GC cells by targeting SOCS genes (SOCS2, SOCS4, and SOCS5) to activate the JAK2/STAT3 signaling pathway (133). Taken together, these studies suggest that targeting the cancer-related signaling pathways is a common miRNA regulation mechanism for miRNAs in GC drug resistance. Understanding the mechanisms of miRNAs in the regulation of the GC signaling pathway may thus provide new insights on therapeutic strategies against GC drug resistance.

### MiRNAs and Drug Efflux in GC

Excessive drug efflux is one of the classical mechanisms of the generation of drug resistance during cancer treatment. Human ATP-binding cassette (ABC) transporters belong to the P-type membrane ATPase superfamily and are closely associated with excessive drug efflux (26). It has been reported that ABC transporters are usually highly expressed in drug-resistant cancer cells, and their overexpression promotes the efflux of excessive intracellular drugs, leading to the impairment of chemotherapeutic effects (134, 135). An increasing number of studies have suggested that miRNAs are involved in the regulation of excessive drug efflux in GC cells by modulating ABC transporters.

Permeability glycoprotein (P-gp), also known as ABCB1 and MDR1, was the first identified ABC transporter closely associated with MDR. Currently, a series of miRNAs, such as miR-103/107, miR-459, miR-361, miR-21, miR-19a/b and mir-129, have been shown to regulate the resistance of GC cells to chemotherapeutic drugs by directly or indirectly targeting P-gp (55, 57, 98, 105, 136, 137). Furthermore, Zhang et al. found that miR-132 is upregulated in Lgr5^+^ GC cells with stem cell-like features, and the overexpression of miR-132 promotes the CDDP resistance of these Lgr5^+^ GC cells. Mechanistically, miR-132 increases the expression of ABCG2 by targeting SIRT1 to downregulate the deacetylation of CREB (138). In addition, Wu et al. reported that the overexpression of miR-129-5p decreases the drug resistance of GC cells by targeting MDR-related ABC transporters, including ABCB1, ABCC5, and ABCG1, whereas the silencing of miR-129-5p shows the opposite effect (139). Shang et al. showed that miR-508-5p overexpression sufficiently reverses the resistance of GC cells to multiple chemotherapeutics and enhances the sensitivity of tumors to chemotherapy by targeting ABCB1 and Zinc ribbon domain-containing 1 (29). Collectively, these findings suggest that miRNAs play a key role in regulating the drug efflux of GC cells, which may provide new insight in the investigation of the roles of miRNA in the drug resistance of GC cells.

### MiRNAs Regulation of Apoptosis in GC Drug Resistance

Inducing the apoptosis of cancer cells is one of the main roles of chemotherapeutic drugs. Therefore, the dysregulation of apoptosis (or its evasion) is commonly characterized as a crucial hallmark of GC drug resistance (140). This may be the result of the aberrant expression of crucial apoptotic proteins or the dysregulation of apoptotic pathways. A large number of studies have shown that miRNAs are involved in the regulation of apoptosis in drug-resistant GC cells by targeting apoptotic proteins or pathways (26, 27, 141).

B-cell lymphoma-2 (BCL-2) family proteins are well-known proteins that regulate mitochondrial apoptosis and are closely associated with chemotherapy resistance (142, 143). Due to their distinct effects on apoptosis, BCL-2 proteins are classified into anti-apoptotic (e.g., Bcl-2, Bcl‐X_L_, and Mcl-1) and pro-apoptotic categories (e.g., Bax, Bad, and Bak) (134, 144). Xia et al. found that miR-15b and miR-16 are downregulated in GC cells. The overexpression of these two miRNAs enhances the sensitivity of GC cells to VCR-induced apoptosis by directly targeting Bcl-2 (66). A study of miR-BART20-5p in Epstein-Barr virus-associated gastric carcinoma showed that miR-BART20-5p promotes the resistance of gastric carcinoma cell line AGS to 5-FU and docetaxel by downregulating the BAD expression (145). Chen et al. showed that miRNA-200c significantly decreases Bax expression and increases Bcl-2 expression by targeting E-cadherin, leading to the inhibition of the resistance of GC cells to 5-FU, PTX, and ADR (146). In our previous work, miR-185 was found to be downregulated in GC tissues and cell lines. The overexpression of miR-185 enhances the sensitivity of GC cells to low-dose CDDP or DOX by targeting apoptosis repressors with a caspase recruitment domain. Conversely, silencing miR-185 inhibits high-dose chemotherapy-induced apoptosis (147). In another study, we showed that miR-633 promotes DOX/CDDP resistance in GC cells by decreasing the expression of Fas-associated proteins with the death domain. Forkhead box O 3 is an upstream regulator of miR-633 that can directly inhibit miR-633 transcription by binding to its promoter region (48). In addition, we also revealed that miR-422a mediates the facilitation effect of lncR-D63785 on the sensitivity of GC cells to DOX (148).

P53 is a well-studied tumor suppressor that mediates major apoptotic pathways to protect cells from malignant transformation. The dysregulation or mutation of p53 contributes to the development of chemotherapeutic drug resistance in cancer treatment (149). Li et al. showed that miR-148a-3p enhances the sensitivity of GC cells to CDDP by promoting mitochondrial fission-induced apoptosis. Mechanistically, miR-148a-3p promotes the activation of P53 to induce DRP1 dephosphorylation by targeting AKAP1, leading to mitochondrial fission and apoptosis in GC cells (150). They also found that the high expression of miR-3174 is closely associated with CDDP resistance in GC cells. MiR-3174 decreases the expression of p53 to inhibit Bax trans-activation by targeting ARHGAP10, thereby suppressing mitochondria-dependent apoptosis (151). In addition, Lee et al. revealed that miR‐193a‐3p triggers the resistance of CD44‐positive GC stem cells against CDDC by regulating the mitochondrial apoptosis pathway. Mechanistically, the high expression of miR‐193a‐3p decreases the expression of Bax, cytochrome C, cleaved caspase 3, and cleaved caspase 9 and increases the expression of Bcl‐XL and Bcl‐2 *via* targeting SRSF2, leading to the enhancement of CDDC resistance in CD44‐positive GC stem cells (152). Furthermore, miR-20a is reported to inhibit apoptosis in CDDC-resistant GC cells by activating the NF-κB pathway. The overexpression of miR-20a upregulates the expression of p65, livin, and survivin by targeting NFKBIB (also known as IκBβ) (132).

### MiRNAs Are Involved in GC Drug Resistance by Modulating Autophagy

Autophagy is a crucial intracellular degradation system that protects cells from the damage of stressors, such as hypoxia and nutrient deprivation (153). It has been reported that chemotherapy-induced autophagy contributes to the acquired drug resistance of cancer cells by helping them escape from deadly cell damage (154). Increasing evidence shows that miRNAs regulate the drug resistance of GC cells by targeting autophagy-related genes (**Table 2**). For instance, the overexpression of miR-30a is found to inhibit chemoresistance-associated autophagy in GC cells by downregulating the expression of light chain (LC)3-II (157). Another study revealed that miR-495-3p overexpression enhances the sensitivity of GC MDR cells to chemotherapy by inhibiting autophagy *via* activating mTOR (a key upstream mediator of autophagy) and targeting GRP78 (160). Moreover, Huang et al. showed that the overexpression of miR-874 enhances the sensitivity of GC cells to chemotherapy by inhibiting the targeting of autophagy occurrence by targeting autophagy-related 16-like 1 (60). Additionally, miR-30e and miR-181a have been shown to enhance the sensitivity of GC cells to chemotherapeutic agents by inhibiting chemo-induced autophagy *via* targeting ATG5 (91, 158). Research on the function of miRNAs in regulating chemotherapy-induced autophagy is currently limited. Further studies are thus required to elucidate its exact mechanisms.

**Table 2:** Regulation of miRNA on autophagy in GC drug resistance.

miRNAs	Alteration	Chemotherapy	Role in autophagy	References
miR-23b-3p	Down	5-FU, VCR, CDDP	miR-23b-3p inhibits autophagy by targeting ATG12 and sensitizes GC cells to chemotherapeutics.	(155)
miR-874	Down	CDDP	miR-874 inhibits autophagy by targeting ATG16L1 and sensitizes GC cells to chemotherapeutics.	(60)
miR-582-5p	Down	oxaliplatin	miR-582-5p inhibits autophagy and sensitizes GC cells to oxaliplatin.	(95)
miR-21	Up	CDDP	miR-21 inhibits autophagy by targeting PI3K/Akt/mTOR pathway and enhances resistance of GC cells to CDDP.	(156)
miR-30a	Down	CDDP	miR-30a inhibits autophagy by targeting P-gp and enhances sensitivity of GC cells to CDDP.	(157)
miR-181a	Down	CDDP	MiR-181a inhibits autophagy by targeting ATG5 and sensitizes GC cells to CDDP.	(158)
miR-30b	Down	CDDP	miR-30b inhibits autophagy by targeting ATG5 and enhances sensitivity of GC cells to CDDP.	(159)
miR-495-3p	Down	5-FU, VCR, CDDP, ADR	miR-495-3p inhibits autophagy by targeting GRP78/mTOR axis and enhances sensitivity of GC cells to chemotherapeutics.	(160)
miR-30e	Down	CDDP	miR-30e inhibits autophagy by targeting ATG5 and enhances sensitivity of GC cells to CDDP.	(91)
miR-148a-3p	Down	CDDP	miR-148a-3p inhibits cyto-protective autophagy by inhibiting RAB12 and mTOR1 activation, and enhances sensitivity of GC cells to CDDP.	(150)
miR-23b-3p	Down	5-FU, VCR, CDDP	miR-23b-3p inhibited autophagy by targeting ATG12 and HMGB2 and sensitized GC cells to chemotherapy.	(65)

### MiRNAs Control Drug Resistance in GC by Modulating Cancer Stem Cell Features

CSCs are recognized as the main cause of chemotherapeutic drug resistance due to their unique characteristics, such as their high DNA repair ability, genomic instability, and overexpression of ABC transporters (161, 162). CSCs are closely associated with the proliferation, metastasis, and recurrence of cancer. An increasing amount of evidence has shown that miRNAs are involved in the regulation of GC drug resistance by affecting CSC properties. For instance, Peng et al. showed that miR-876-3p is downregulated in CDDP-resistant GC cells and its low expression is closely associated with the CDDP resistance of GC. MiR-876-3p confers sensitivity to CDDP-resistant GC cells. Mechanistically, the overexpression of miR-876-3p downregulates the expression of Sox-2, Oct-4, CD133, and CD44 by targeting TMED3, thereby inhibiting the stem cell-like features of GC cells (163). Zeng et al. revealed that the expression of miR-145 is decreased in GC cells. The overexpression of miR-145 enhances the sensitivity of GC cells to chemotherapeutic drugs by inhibiting the stem-like properties of GC *via* directly targeting CD44 (164). Lee et al. reported that miR‐193a‐3p overexpression promotes the resistance of CD44‐positive gastric CSCs to CDDC by modulating the mitochondrial apoptosis pathway *via* targeting SRSF2 (152). In addition, some miRNAs, such as miRNA-106b, miR‐196a‐5p, and miR-26a, are also found to regulate GC stem-like cell properties (165–167), indicating their great potential in regulating the drug resistance of GC.

### MiRNAs and EMT in GC Drug Resistance

EMT is a morphogenetic process that changes epithelial cells from a pebble-like phenotype to a fibroblast-like phenotype, which endows cells with migratory and invasive properties. During the EMT process, the mesenchymal markers (e.g., Vimentin, N-Cadherin) are upregulated, whereas the epithelial markers (e.g., E-cadherin) are downregulated (168). It has been reported that the aberrant activation of EMT contributes to the development of drug resistance in cancer by enabling the conversion of non-CSCs into CSCs (169). An increasing number of studies have suggested that miRNAs play crucial roles in drug resistance by directly targeting the EMT process (**Table 3**). For instance, Wang et al. showed that miR-30a is downregulated in CDDP-resistant GC cells. The overexpression of miR-30a increases the CDDP sensitivity of GC cells by inhibiting EMT *via* downregulating the Snail and Vimentin levels. GC cells with miR-30a knockdown show decreased sensitivity to CDDP (173). Li et al. revealed that miR-204 is downregulated in 5-FU-resistant GC cells, with the epithelial markers (E-cadherin) decreased and the mesenchymal markers (N-cadherin, Fibronectin, Twist, and Snail) increased. The overexpression of miR-204 sensitized GC to 5-FU by inhibiting TGF-β-induced EMT *via* targeting TGFBR2 (36). ZEB1 is a crucial EMT-inducing transcription factor. Wang et al. found that miR-574-3p overexpression inhibits the CDDP resistance of GC cells. Mechanistically, the overexpression of miR-574-3p increases E-cadherin expression and decreases vimentin expression by targeting ZEB1 *via* binding to its 3’-UTR, thereby enhancing the sensitivity of GC cells to CDDP (171). Moreover, miR‐200c overexpression is found to enhance the trastuzumab sensitivity of GC cells by inhibiting ZEB1 and ZEB2 (130). Additionally, Wang et al. showed that the overexpression of exosomal miR-155-5p derived from PTX-resistant GC cells induces EMT progress and enhances drug resistance in PTX-sensitive GC cells by targeting GATA binding protein 3 (GATA3) and tumor protein p53-inducible nuclear protein 1 (TP53INP1) (103). Collectively, these findings suggest that miRNAs are crucial regulators determining the fate of drug-resistant GC cells by targeting the EMT process.

**Table 3:** The role of miRNA-mediated EMT in GC drug resistance.

miRNAs	Alteration	Chemotherapy	Role in EMT	References
miR-30a	Down	CDDP	miR-23b-3p inhibits EMT by upregulating E-cadherin and downregulating N-cadherin *via* targeting P-gp and sensitizes GC cells to CDDP.	(38)
miR-200c	Down	trastuzumab	miR-200c inhibits EMT by targeting ZEB1 and ZEB2 and sensitizes GC cells to trastuzumab.	(130)
miR-155-5p	Up	PTX	miR-155-5p promotes EMT by targeting GATA3 and TP53INP1 and enhances resistance of GC cells to PTX.	(170)
miR-574-3p	Down	CDDP	miR-23b-3p inhibits EMT by upregulating E-cadherin and downregulating vimentin *via* targeting ZEB1 and sensitizes GC cells to CDDP.	(171)
miR-204	Down	5-FU	miR-204 inhibits EMT by upregulating mesenchymal markers and downregulating epithelial marker *via* targeting TGFBR2 and sensitizes GC cells to 5-FU.	(36)
miR-17	Up	CDDP, 5-FU	miR-155-5p promotes EMT by targeting DEDD and enhances resistance of GC cells to chemotherapeutics.	(172)
miR-27a-5p	Down	DOX	miR-23b-3p inhibits EMT by regulating MAPK and AKT pathways *via* targeting APEX1 and sensitizes GC cells to DOX.	(127)
miR-95-3p	Up	CDDP	miR-155-5p promotes EMT by regulating PI3K/AKT pathway targeting EMP1 and enhances resistance of GC cells to chemotherapeutics.	(76)

### MiRNAs Influence Cell Cycle Progression in GC Drug Resistance

Cell cycle alteration is one of the main processes involved in drug resistance. The dysregulation of the cell cycle may lead to drug resistance (28, 174). Therefore, targeting cell cycle progression factors may provide new insight into therapeutic strategies for cancer. Many miRNAs have been proven to be involved in GC drug resistance by regulating cell cycle progression. Cyclin D1 is a major regulator of cell cycle progression that governs the entrance of a cell from the G1 phase into S (175). Hu et al. revealed that miR-449a is downregulated in both GC tissues and cell lines. The overexpression of miR-449a suppresses proliferation and promotes CDDP-mediated apoptosis in GC cells. They further showed that miR-449a reduced the percent of S phase cells and increased the percent of G1/G0 phase cells by targeting BCL2 and cyclin D1, leading to the enhancement of the CDDP sensitivity of GC cells (176). In another study, Jiang et al. showed that miR-623 is downregulated in both GC tissues and cell lines. The overexpression of miR-623 enhances the sensitivity of GC cells to 5-FU by targeting Cyclin D1 (42). In addition, some miRNAs, such as miR-1301-3p, miR-3663-3p, and miR-34a, are also found to regulate cell cycle progression by targeting cyclin D1, indicating their potential role in the drug resistance of GC (177–179). F-box and WD repeat domain-containing 7 (FBXW7) is a classical tumor suppressor that promotes the ubiquitination and degradation of several oncoproteins, such as Cyclin E, c-MYC, and c-JUN (180). Zhou et al. demonstrated that miR-223 is up-regulated in CDDP-resistant GC cells. The knockdown of miR-223 enhances the sensitivity of resistant GC cells to CDDP by inducing cell arrest in the G0/G1 phase. Mechanistically, miR-223 modulates the cell cycle of GC cells by targeting FBXW7 *via* binding to its 3’-UTR, thus affecting the sensitivity of the GC cells to CDDP (181). The early region 2 binding factor (E2F) family of transcription factors are well-studied major transcriptional regulators of cell cycle-dependent gene expression (182). Wen et al. showed that miR-26a is downregulated in CDDP-resistant GC cells. Function analysis demonstrated that miR-26a improves the sensitivity of GC cells to CDDP by targeting E2F2 and NRAS (183). Another study revealed that the overexpression of miR-34a enhances the sensitivity of GC cells to PTX by targeting E2F5 (106). Taken together, these findings provide new insights into the mechanisms of GC drug resistance mediated by miRNAs involved in cell cycle regulation, which may improve chemotherapy effectiveness.

### MiRNAs and T Cells in GC Drug Resistance

T cells are the major effector cells in tumor immunity and produce cytokines in immune responses to mediate inflammation and regulate other types of immune cells (184, 185). It has been reported that the immune escape and immune tolerance induced by the dysregulation of cytotoxic T cell activity are closely associated with drug resistance in cancer (186). Some studies suggested that miRNAs are involved in drug resistance by regulating T cell activity in multiple cancer types. Xu et al. demonstrated that the restoration of miR-424 (322) expression in chemoresistant ovarian cancer cells could activate the T-cell immune response by regulating the production of CD8^+^ T, MDSC, and Treg cells *via* targeting PD-L1, resulting in the reversal of drug resistance (187). Qian et al. showed that the overexpression of miR-101 inhibits cell proliferation and invasion and induces apoptosis by targeting Notch1 in T-cell acute lymphoblastic leukemia (T-ALL) cells. Further analysis revealed that miR-101 enhances the sensitivity of T-ALL cells to the chemotherapeutic agent ADR (188). MiRNAs can also regulate GC progression by influencing T cells. For instance, miR−140 plays an anti-tumoral effect in GC by increasing cytotoxic CD8+ T cell and reducing myeloid-derived suppressive and regulatory T cell infiltration (189). Exosomal miRNA-16-5p derived from M1 macrophages inhibits GC development through activation of T cell immune response *via* PD-L1. These findings strongly indicate that miRNAs may participate in the development of GC drug resistance by regulating T cells. Thus, in-depth studies are required to elucidate the mechanism of miRNAs in regulating T cells in GC drug resistance, which may provide new insights into the development of miRNA-based therapeutics strategies in GC.

## Clinical Applications of Drug Resistance-Related MiRNAs in GC

### MiRNAs as Diagnostic and Prognostic Biomarkers

Currently, most GC patients are still diagnosed at an advanced stage with poor prognosis due to the lack of an effective approach for early detection and prognostic evaluation in clinical practice. Some protein biomarkers, such as CEA, uPA, and CA 19-9, have been applied in clinic, but the low specificity and sensitivity of these biomarkers limit their further utilization (190–192). Therefore, it is important to screen and identify novel biomarkers for the early detection and prognostic evaluation of GC patients, particularly those demonstrating a poor response to chemotherapy.

An increasing number of studies suggest that miRNAs possess great potential to be biomarkers for the diagnosis and prognosis of GC patients. In a latest clinical trial consist of 5248 GC and control subjects, So et al. developed a clinical diagnostic assay for GC from a high-risk population based on a serum 12-miRNA biomarker panel. In the discovery cohorts, the 12-miRNA panel has an area under the curve (AUC) of 0.93 in discriminating early GC patients from normal controls. Excitingly, the AUC value also reached 0.92 in the verification cohorts. Further prospective study revealed that the AUC value for the 12-miRNA panel was 0.848, which is higher than HP serology (0.635), PG 1/2 ratio (0.641), PG index (0.576), ABC method (0.647), CEA (0.576), and CA19-9 (0.595). Moreover, the overall sensitivity of the 12-miRNA assay was 87.0% at specificity of 68.4% (193). These data strongly suggest that serum 12-miRNA panel is a promising biomarker with higher GC diagnostic accuracy than traditional serum-based biomarkers. In another study consist of 354 GC patients, Shimura et al. identified a miRNA-based signature (including miR-30a-5p, -134-5p, -337-3p, -659-3p, and -3917), which can be used as a biomarker to identify peritoneal metastasis (PM) in GC patients. The AUC value for the combination miRNA signature was 0.82 in distinguishing GC patients with versus without PM. In an independent validation cohort, the AUC value reached 0.74 (194).

It has been reported that miRNAs are involved in drug resistance and may serve as promising biomarkers for predicting drug resistance and prognosis in GC (26) (**Table 4**). For instance, Jin et al. found that miR-3180-3p is significantly upregulated and miR-124-3p is downregulated in both drug-resistant GC patients and cell lines. The combination of the two miRNAs can effectively distinguish drug-resistant GC patients from drug-sensitive GC patients (AUC = 0.946 ± 0.023, p < 0.001), indicating their potential as serum-based biomarkers in predicting the therapeutic benefit of CDDP in GC (204). Ji et al. showed that miR-374a-5p is upregulated in GC serum, and its upregulation predicts poor prognosis of GC patients. The overexpression of miR-374a-5p promotes the drug resistance of GC cells by targeting Neurod1, indicating the great value of miR-374a-5p as a biomarker for GC diagnosis and prognosis (195). Besides, miR-1229-3p is significantly upregulated in the plasma of GC patients, and its upregulation is an independent poor prognostic factor for recurrence-free survival. The overexpression of miR-1229-3p promotes the significant drug resistance of GC cells to 5-FU both *in vitro* and *in vivo*. This data indicate that plasma miR-1229-3p can serve as a clinically useful biomarker for predicting drug resistance to 5-FU in GC patients (200).

**Table 4:** Drug resistance-related miRNAs as diagnostic and prognostic biomarkers in GC.

miRNAs	Alteration	Potential values	References
miR-374a-5p	Up	High level of miR-374a-5p predicts poor prognosis and poor response to chemotherapy.	(195)
miR-15a-5p	Up	High level of miR-15a-5p predicts poorer survival and poor response to chemotherapy.	(196)
miR-567	Down	Low level of miR-567 predicts poor response to chemotherapy.	(37)
Let-7a	Down	Low level of Let-7a predicts poor response to chemotherapy.	(197)
miR-363	Up	High level of miR-363 predicts poorer survival and poor response to chemotherapy.	(198)
miR-582-5p	Down	Low level of miR-582-5p predicts poorer survival and poor response to chemotherapy.	(95)
miR-9-5p, miR-9-3p, miR-433-3p	Up	Low level of miR-9-5p or combination of high level of miR-9-5p, miR-9-3p, and miR-433-3p predicts poorer survival and poor response to chemotherapy.	(199)
miR-1229-3p	Up	High level of miR-1229-3p predicts poor response to chemotherapy.	(200)
miR-27a	Up	High level of miR-27a predicts poorer survival and poor response to chemotherapy.	(201)
miR-21	Up	High level of miR-21 predicts poorer survival and poor response to chemotherapy.	(202)
miR-16	Down	Low level of miR-16 predicts poor response to chemotherapy.	(203)
miR-508-5p	Down	Low level of miR-508-5p predicts poorer survival and poor response to chemotherapy.	(29)
miR-23b-3p	Down	Low level of miR-23b-3p predicts poorer survival and poor response to chemotherapy.	(65)

In addition, Huang’s group found that partial response rates of GC patients with high miRNA27a expression and low miRNA27a expression to fluoropyrimidine‐based chemotherapy to be 7.7% and 25.9%, respectively (P = 0.018). GC patients with high miRNA27a expression have a significantly worse overall survival (OS) than those with lower miRNA27a expression (P = 0.024). These results suggest that miRNA27a is a promising biomarker for predicting resistance to fluoropyrimidine‐based chemotherapy and a novel prognostic biomarker for metastatic or recurrent GC (201). MiR-21 in both tumor tissue and plasma is significantly upregulated in drug-resistant GC patients compared to the drug-sensitive GC patients (p < 0.001). ROC analysis showed that the expression of miR-21 in tissue distinguished drug-resistant GC patients from drug-sensitive GC patients, with an AUC of 0.830, 88.0% sensitivity, and 68.7% specificity; moreover, the expression of miR-21 in tissue plasma distinguished drug-resistant GC patients from drug-sensitive GC patients, with an AUC of 0.759, 52.0% sensitivity, and 88.1% specificity. Moreover, GC patients with high miR-21 expression exhibit shorter OS time than patients with low miR-21 expression, indicating that miR-21 might be a promising biomarker for identifying metastatic GC with drug resistance (202).

In a recent study of Jin et al., they found that miR-9-3p, miR-9-5p, miR-146a-5p, and miR-433-3p were closely associated with chemotherapy responses in GC patients and cells. MiR-9-5p distinguished drug-resistant GC patients from drug-sensitive GC patients with an AUC of 0.856 and p < 0.0001; moreover, the combination of miR-9-3p, miR-146a-5p, and miR-433-3p distinguished drug-resistant GC patients from drug-sensitive GC patients, with an AUC of 0.915, and p < 0.0001, suggesting the great potential of miR-9-5p and the combined group (miR-9-3p, miR-146a-5p, and miR-433-3p) as serum-based biomarkers distinguishing drug-resistant GC (199). These findings strongly suggest that miRNAs possess great potential as valuable biomarkers for predicting the drug response and prognosis of GC. However, more in-depth studies are required to overcome their limitations in clinical application using high-quality samples and larger patient cohorts.

### Therapeutic Potential of MiRNAs in GC Drug Resistance

A single miRNA can simultaneously modulate several genes by targeting different mRNAs, and one gene can also be regulated by multiple miRNAs, indicating that miRNAs possess great potential as effective therapeutic targets or therapeutic agents in cancer treatment. Aberrantly expressed miRNAs have been shown to play crucial roles in the development of GC drug resistance. Therefore, correcting these miRNA deficiencies by either antagonizing or restoring miRNA functions may provide new insights into the development of therapeutic strategies for reversing GC drug resistance. Currently, the main miRNA-based therapies contain nanoparticles, miRNA mimics, miRNA inhibitors, locked nucleic acid, decoy vectors, DNA sponge, antagomiRs, and small compounds (205–208). Wang et al. found that exosomes can act as nanoparticles to deliver anti-miR-214 to reverse the resistance of GC cells to CDDP. Exosome-delivered anti-miR-214 is maintained at a stable level in the blood and downregulated the expression of miR-214 in the tumor tissues of mice, thereby enhancing the sensitivity of refractory GC to CDDP (209). Ghasabi et al. showed that miR-200c mimics significantly reduced the resistance of GC cells to CDDP and increased CDDP-induced apoptosis by targeting RhoE (210). Ji et al. revealed that the knockdown of miR-374a-5p using miR-374a-5p inhibitor repressed the resistance of GC cells to oxaliplatin and promoted cell apoptosis induced by oxaliplatin (195). In addition, miR-21 has been found to confer CDDP resistance in GC. The miR-21 inhibitor sensitized CDDP-resistant GC cells by inducing autophagy *via* the PI3K/Akt/mTOR pathway (156). These findings support that miRNA could become effective therapeutic target or therapeutic agent reversing GC drug resistance. However, miRNA-based therapies have not been translated into GC clinical treatment. More detailed and in-depth studies are still required to investigate the roles of miRNAs in GC drug resistance.

## Conclusion and Perspective

Chemotherapy is still the first-line standard treatment option for cancer patients. However, the development of drug resistance results in chemotherapy failure and cancer recurrence, both of which seriously threaten patients’ health and lives. In recent years, increasing numbers of miRNAs have been found to be aberrantly expressed in drug-resistant GC tissues. Most have been shown to be involved in the regulation of GC sensitivity or resistance to chemotherapeutic agents by influencing various aspect of GC cell function, including drug efflux, apoptosis, autophagy, EMT, CSCs, and cell cycle. Moreover, a complex cross-talk network has been observed between miRNAs and several drug resistance-related signaling pathways in GC (**Figure 2**).

**Figure 3:**
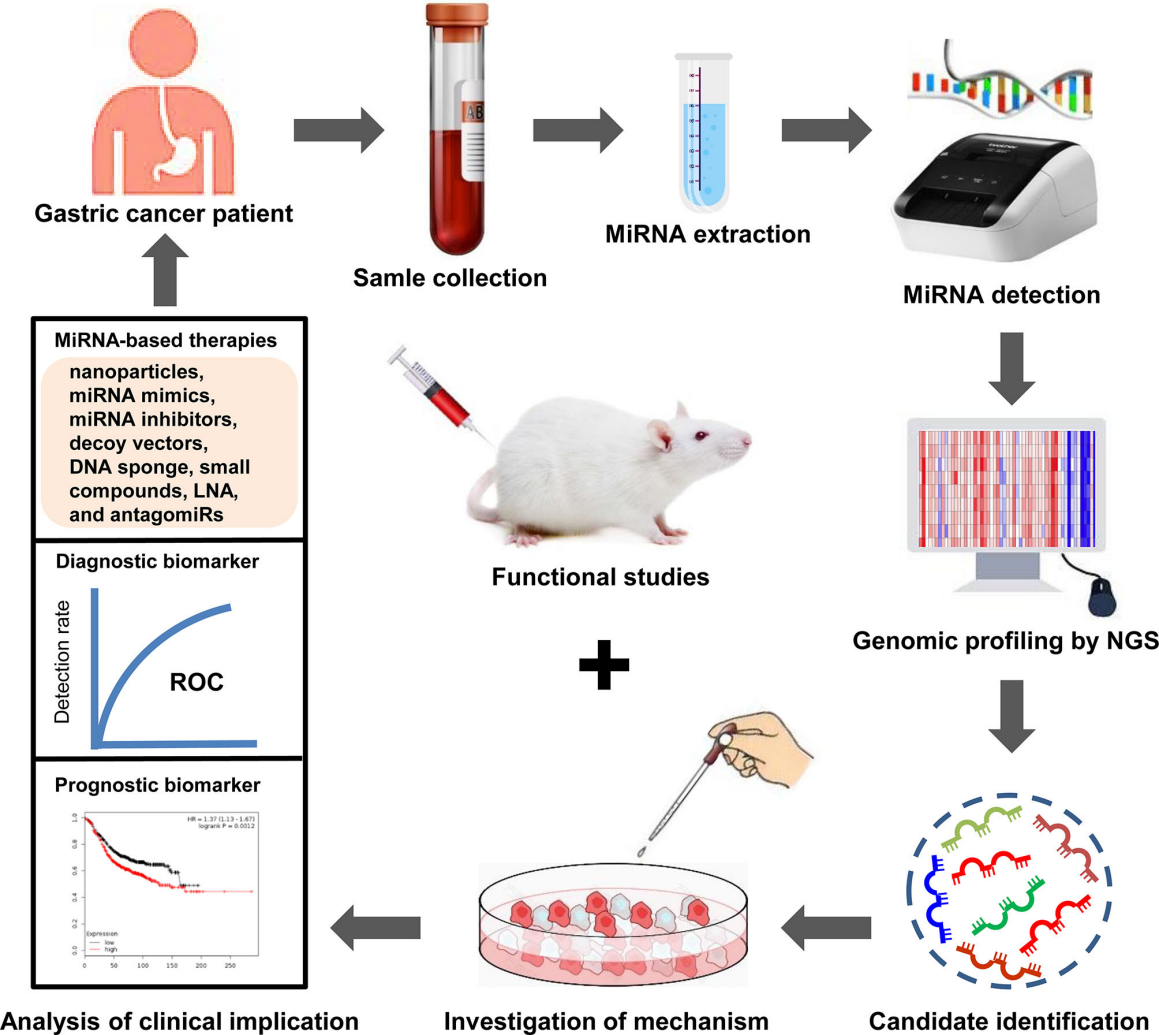
Clinical implications of miRNAs in GC drug resistance. MiRNAs are enriched in blood samples from drug resistant-GC patients. Dysregulated miRNAs are identified through high-throughput sequencing. Subsequently, the mechanisms of dysregulated miRNA in the drug resistance of GC are investigated using cell models. The functions of dysregulated miRNA in GC drug resistance are investigated using animal models. Next, the dysregulated miRNAs that can act as diagnostic or prognostic biomarkers for drug resistant-GC patients are selected. Moreover, targeting specific miRNAs may produce reliable therapeutic effect. Finally, GC patients with drug resistance receive individualized precision treatment based on these effective strategies.

MiRNAs have been shown to be crucial regulators of several oncogenic pathways during GC progression. Moreover, miRNAs are small in size. Thus, it is easier to design specific drugs targeting them or deliver them to target tissues. These features strongly suggest that miRNAs are ideal therapeutic targets for GC patients. In the other hand, miRNAs in plasma/serum are well protected from RNases, they remain stable under harsh conditions (211), which endows them with great potential as biomarkers for the early diagnosis and prognostic evaluation of GC patients. Therefore, the identification of drug resistance-related miRNAs and the investigation of their mechanisms in GC progression are crucial for designing novel effective therapeutic strategies for GC patients, especially those exhibiting a poor response to chemotherapy. In addition, the combination of miRNAs with existing chemotherapeutic agents may provide a new option for maximizing therapeutic effects and improving clinical outcomes in GC patients (**Figure 3**). However, there are still some challenges in translating the findings on miRNA-mediated drug resistance in GC into clinical utilization, such as side effects, minimized off-target effects, and the modes of targeted delivery. Nevertheless, miRNA-based therapies may provide promising therapeutics for GC patients to overcome drug resistance and increase their survival in the future. Further studies are needed to clarify the exact mechanism of miRNAs in the regulation of GC drug resistance using large-scale clinical trials.

In summary, recent studies have shown that miRNAs possess the great potential to be effective therapeutic targets and promising biomarkers for predicting drug resistance and prognosis in GC treatment. In-depth understanding of their mechanism in GC progression may facilitate the design of potential therapeutic strategies that can be used to reverse drug resistance in GC patients. However, overcoming resistance to chemotherapy still remains a big challenge. Continuous efforts are required to develop miRNA-based therapies that can provide novel therapeutic options and thus improve the clinical outcomes of GC patients in the future.

## Author Contributions

YL: Writing- Conceptualization, Original draft preparation, Writing - Review and Editing, Funding acquisition. XA: Data Curation, Writing - Review and Editing. GJ: Data Curation. YZ: Data Curation. WY: Data Curation. JW: Writing - Review and Editing. All authors contributed to the article and approved the submitted version.

## Funding

All authors are supported by Qingdao Medical College, Qingdao University. This work was funded by the National Natural Science Foundation of China (81802822), the China Postdoctoral Science Foundation (2018M642607), the Natural Science Foundation of Shandong Province (ZR2021MC189), and the Qingdao Applied Basic Research Project (19-6-2-50-cg).

## Conflict of Interest

The authors declare that the research was conducted in the absence of any commercial or financial relationships that could be construed as a potential conflict of interest.

## Publisher’s Note

All claims expressed in this article are solely those of the authors and do not necessarily represent those of their affiliated organizations, or those of the publisher, the editors and the reviewers. Any product that may be evaluated in this article, or claim that may be made by its manufacturer, is not guaranteed or endorsed by the publisher.
